# Factors Influencing Continued Usage Behavior on Mobile Health Applications

**DOI:** 10.3390/healthcare10020208

**Published:** 2022-01-21

**Authors:** Pei Wu, Runtong Zhang, Xiaomin Zhu, Manlu Liu

**Affiliations:** 1 School of Economics and Management, Beijing Jiaotong University, Beijing 100044, China; peiwwu@bjtu.edu.cn; 2 School of Mechanical, Electronic and Control Engineering, Beijing Jiaotong University, Beijing 100044, China; xmzhu@bjtu.edu.cn; 3 Rochester Institute of Technology, Saunders College of Business, Rochester, NY 14623, USA; manluliu@saunders.rit.edu

**Keywords:** mHealth apps, e-satisfaction, continued usage intention, continued usage behavior, UTAUT2

## Abstract

(1) Background: As people pay more attention to health, mobile health applications (mHealth apps) are becoming popular. These apps offer health services that run on mobile devices to help improve users’ health behaviors. However, few studies explore what motivates users to continue to use these apps. This study proposes antecedents influencing users’ electronic satisfaction (e-satisfaction) and their continued behaviors of using mHealth apps. Based on the extended Unified Theory of Acceptance and Use of Technology (UTAUT2), this study constructs a research model including perceived reliability and online review to predict the continued usage behavior on mHealth apps in China; (2) Methods: We conduct an online survey to collect data from participants who have used mHealth apps. This study receives 327 valid responses and tests the research model using the partial least squares structural equation model approach; (3) Results: Our results find that antecedents positively affect continued usage intention through the mediation role of e-satisfaction with mHealth apps. Interestingly, this study reveals that habit positively affects the continued usage behavior and moderates the effect of e-satisfaction and continued intention of using mHealth apps; (4) Conclusions: This study presents theoretical implications on the extended UTAUT2 and provides practical implications understanding of managing mHealth apps in China.

## 1. Introduction

The speedy evolutions of information technology (IT) have encouraged mobile devices to become a broad part of daily life. The widespread mobile device usage contributes to the integration of health and mobile services [1]. mHealth apps have changed the way health information acquiring and improved the efficacy of services in the healthcare field [2,3]. Zion Market Research [4] proposes that the mHealth apps market will be more than USD 111 billion by 2025. However, people use mHealth apps unfrequently, likely because these apps are perceived as unreliable [5]. The development of mHealth apps depends on their attracting and retaining users. Understanding users’ continued usage behavior is essential for the success of mHealth apps. In this study, mHealth apps refer to applications installed on mobile devices that provide users with health information and have simple reminders and health data tracking functions to effectively promote users’ self-health behaviors and improve the efficiency of health services and the accessibility of health information.

Though mHealth apps thrive, people rarely continue to use such apps after the initial acceptance [5]. Among the empirical studies on users’ continued use of health technology, researchers have explored the impact of factors on the continued behavior of using health apps. For example, Cho [6] identified the effects of perceptual and emotional factors on motivating continuous using health apps relying on the post-acceptance model and the technology acceptance model; based on the social cognitive theory, Kim and Han [7] examined the effects of health technology self-efficacy, self-evaluative outcome expectations, self-regulation, and privacy risk on older users’ continued intention to use health apps. Recently, one previous study proposed a research model including context and contents values to explore the intention of users over the age of 40 in using health apps on mobile phones [8], another study applied the expectation-confirmation model and the investment model to explore the effects of perceived usefulness, satisfaction, and commitment on continuance intention to use health apps [9]. However, few studies explored the effects of factors on the intention and behavior of keeping to use mHealth apps based on the extended UTAUT2.

In addition, previous studies have conducted and proposed key factors affecting users’ adoption intentions in using mHealth, such as performance expectancy, effort expectancy, social influence, and facilitating conditions [10,11]; self-efficacy and response-efficacy [12]. However, rarely studies explore the mediation role of user e-satisfaction among antecedents and continued intention of using mHealth apps. E-satisfaction refers to users’ satisfaction with the experience on mHealth apps. If the effects of using mHealth apps meet and exceed users’ expectations, users may be satisfied with mHealth apps and willing to have a continued usage intention. Furthermore, although user’s habit is a critical factor in affecting continued intention and behavior when using mobile food ordering apps [13], few studies discuss the moderation role of habit in mHealth apps continues to use. To address the above research gaps, this study focuses on the following research questions:

RQ 1: Which factors influence intention to continue to use mHealth apps?
RQ 2: How do various factors influence the continued usage of mHealth apps?

The research objective of this paper is to explore the factors that affect the users’ intentions to keep using mHealth apps, and their continued behaviors that are affected by the intentions. First, based on the extended UTAUT2, this study identifies the determinants of intention to continue to use mHealth apps through the mediation role of e-satisfaction. Second, this study attempts to explore the impacts of the habit of using mobile apps on the continued usage behavior and innovatively analyzed the moderating effect of habits on the relationship between e-satisfaction and continued usage intention. Third, this study examines the effects of antecedents on users’ e-satisfaction with mHealth apps through integrating the perceived reliability and online review in the extended UTAUT2.

The contributions of this study include three aspects. First, through exploring the effects of antecedents on users’ e-satisfaction with mHealth apps, this study makes an original contribution to integrating e-satisfaction into the UTAUT2 in the context of mHealth apps. Users’ e-satisfaction, as a positive attitude, has significant effects on the continued intention of using mHealth, which promotes the success of mHealth apps. Second, the characterization of e-satisfaction is important for our increased understanding of the effects of antecedents on continued usage intentions. This study identifies the mediation role of e-satisfaction on the relationships between antecedents and continued usage intentions. Third, the empirical work presents here provides one of the first investigations into how habits influence the relationship between e-satisfaction and continued usage intentions. The results of this study make a major contribution to research on the continued behavior of using mHealth apps by demonstrating the moderating effect of the habit of using mHealth apps on the positive association between e-satisfaction and continued usage intentions.

The rest of the paper is shown as follows. First, this study presents the literature review. Second, based on the UTAUT2, this study provides the hypotheses and research model. Third, we describe our research methods including four sections (development of the questionnaire, data collection, and data analysis). Fourth, we introduce our results from four aspects including measurement model analysis and structural model analysis. Finally, we discuss the theoretical implications, practical implications, and limitations of this study.

## 2. Literature Review

The popularity of mobile devices has promoted the development of various apps that provide people with a wide range of services [14,15]. Considering that people are paying more attention to health, mHealth apps have developed into an important tool for users to solve health problems [16,17]. Currently, in developing countries, mHealth apps can offer faster ways to share information related to patients’ diseases and provide a wealth of professional health information [18,19]. Based on the analysis of functions that support health behavior change techniques, tracking health data is the most important feature in mHealth apps [20]. Setting customized goals, notifications and reminders, and access to health information are the second most important features for users [20]. In addition, mobile technologies provide users with adaptive, low-cost, and easily accessible self-management interventions [21,22,23].

People most often learn of mHealth apps from relatives or friends rather than from health professionals and existing studies have found that social influence positively affects behavioral intention to use mHealth [12]. Previous studies have found that people who use mHealth apps continuously for a long time can effectively improve their overall health fitness [7,20]. Though the commitment to mHealth apps is beneficial, the abandoning phenomenon of such apps is common [20]. People initially downloaded mHealth apps but later uninstalled them [9]. The reason for this phenomenon may be that users who are not positively affected by using mHealth apps have no beliefs in performance expectancy, social influence, facilitating condition and perceived reliability of these apps [12,24].

Previous studies discussed the effects of different factors on the continued use of mobile apps, such as perceived usefulness, satisfaction, and commitment [6,9]; price value, habit, online review, and e-satisfaction [13]. In the context of mHealth, the existing evidence from the effects of performance expectancy and effort expectancy on users’ behaviors of using mHealth services are inconsistent. For example, Mohammad et al. [12] found that effort expectancy did not affect behavioral intention and perceived reliability of services quality significantly affects individuals’ intentions to accept mHealth services; Hoque and Sorwar [25] found that effort expectancy significantly affects users’ behavioral intention. In addition, satisfaction with mobile apps and attitude toward such apps has proved to be a critical factor of influencing the continued intention and behavior of using these apps [13,26,27]. For example, one study found that satisfaction has mediation effects on the key factors (perceived usefulness, perceived ease of use, flow experience, and behavioral change techniques) of continued intention to use mHealth apps [26]; another study revealed that attitude positively influences intention to use mHealth apps from protection motivation and network externality perspectives [27].

## 3. Theoretical Background and Hypotheses Development

UTAUT is developed by integrating eight user acceptance models that include the technology acceptance model (TAM), reasoned action theory, motivational model, planned behavior theory, the model combining the TAM and planned behavior theory, personal computer utilization model, innovation diffusion theory, and social cognitive theory [28]. Based on UTAUT, previous studies have assessed people’s acceptance of emerging technology in developing countries [29]. For example, an empirical study has explored the intention of the elderly to use emerging health apps [30]. Some researchers have focused on the intention of using mobile technology [31,32]. Venkatesh et al. [33] extended hedonic motivation, price value, and habit in UTAUT affecting individuals’ acceptance of information technology and found that UTAUT2, compared with UTAUT, increased the interpretation of individuals’ usage intention and behavior. Based on UTAUT2, previous studies have examined the continued intention of mobile technology. For example, Alalwan [13] investigated the positive effects of online review, performance expectancy, hedonic motivation, and price value on e-satisfaction and continued intention of using mobile food order apps; Beh et al. [34] explored the determinants of smartwatches usage and provided useful insights into drivers of the emerging technology for fitness and health monitoring.

UTAUT is regarded as a theoretical framework to study the continued usage behavior of mHealth apps and proposes antecedent factors that influence users’ intentions for keeping to use mHealth apps. Compared with UTAUT, UTAUT2 greatly improves the interpretation of individuals’ usage behavior and is developed to illuminate users’ continued usage behavior of mHealth apps [33,35]. Price value is important as people must bear the costs related to purchasing mobile devices and mHealth services [33]. Habit in technology use is a significant factor in predicting technology acceptance [33]. Online reviews have drawn widespread attention in mobile technology literature as review visibility is quite significant and meaningful in the consumption process [36]. Perceived reliability is the potential determinant of users’ willingness to adopt mHealth service innovations [12,37]. However, few studies focus on the effects of perceived reliability and online review on the continued intention of using mHealth apps and the mediating role of e-satisfaction in the theoretical framework of UTAUT2.

**Figure 1:**
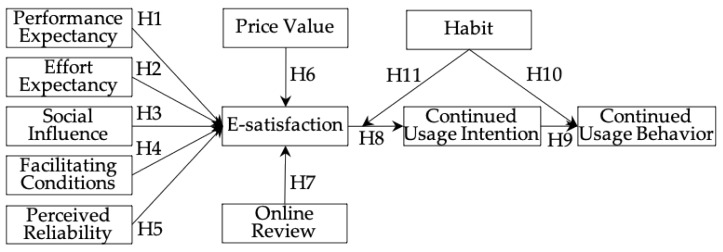
Research model.

To understand the theoretical framework, this study proposes a research model (see Figure 1) to address the research questions associated with the continued usage behavior of mHealth apps. This study integrates online review and perceived reliability into UTAUT2 to tailor it to the research of individuals’ continued acceptance of mHealth apps. This study focuses on the UTAUT2 to explore the effects of antecedents on users’ e-satisfaction with mHealth apps and the mediation role of e-satisfaction on the relationship between antecedents and continued intention. In addition, this study explores the moderation role of habit on the relationship between e-satisfaction and continued usage intentions.

### 3.1. Performance Expectance

Emerging technologies usage can provide individuals with benefits in conducting certain activities, which is the definition of performance expectancy [33]. People will be more willing to adopt emerging technologies if they believe that technologies have advantages [38]. When people use mHealth services, performance expectancy represents their expectations in the case of demand environments [12] and is the main driver of their satisfaction with the environments. Moreover, mHealth apps are the application of mobile technologies in healthcare and provide convenience for the communication among users due to the characteristics, such as mobility and flexibility. People using mHealth apps can acquire comprehensive health information and consult professional physicians at any time and any place [39]. People can choose professional physicians for health consultation without location movement through these apps. Using mHealth apps is particularly convenient in consideration of issues such as queuing for registration, transportation costs, and long waiting times for health consultations. In other words, if users have high utilitarian value when using such innovative mHealth apps, they may be satisfied [13].

**Hypothesis** **1** **(H1).** *Performance expectancy positively affects users’ e-satisfaction with mHealth apps*.

### 3.2. Effort Expectance

Effort expectancy explains the ease of usage behavior of technologies, which is one of the significant components in researches on technologies acceptance [33,40]. Effort expectancy is closely related to users’ satisfaction [13,41] and has an effect on satisfaction in the field of online learning [13,42]. In the context of mHealth apps, people complete the evaluation of health information without any assistance from healthcare staff. People spend time and effort to reflect the complexity and ease of using mHealth apps. Peoples’ satisfaction with mHealth apps can be determined by the ease and simplicity of using them.

**Hypothesis** **2** **(H2).** *Effort expectancy positively affects users’ e-satisfaction with mHealth apps*.

### 3.3. Social Influence

The definition of social influence is the extent to which people are influenced by essential others (family and friends) who recommend using emerging technologies [33]. Social influence is one of the most significant factors related to individuals’ usage and rejection of mHealth apps. Most people are not completely familiar with mHealth apps and are mainly influenced by others’ opinions and attitudes, such as those of friends, relatives, and colleagues [13,35,43]. Moreover, when judging users’ satisfaction with mHealth apps, it is important to point out that they are susceptible to the effect of other users. People may also gain social recognition from others, which will increase their social value and their satisfaction with mHealth apps [44]. In addition, social influence significantly impacts users’ satisfaction with mobile apps [45].

**Hypothesis** **3** **(H3).** *Social influence positively affects users’ e-satisfaction with mHealth apps*.

### 3.4. Facilitating Conditions

Facilitating conditions refer to peoples’ perceptions of the technological infrastructures to support using emerging technologies [28]. Facilitating conditions positively affect peoples’ intention of using smartphones for health services [46]. Peoples’ experience in mHealth apps and the degree of satisfaction depend on the technical infrastructure and human support available at their request [13]. The role of personnel support in services is essential to ensure the delivery of high-quality services. Considering the important role of convenience, if users obtain a sufficient level of technology, organization, infrastructure, and personnel support when using mHealth apps, they may have simpler and more convenient experiences, thereby making them more satisfied with such apps [13]. Moreover, prior studies have proved that close relationships exist between facilitating conditions and satisfaction in healthcare [47,48].

**Hypothesis** **4** **(H4).** *Facilitating conditions positively affect users’ e-satisfaction with mHealth apps*.

### 3.5. Perceived Reliability

Perceived reliability refers to the extent to which people believe emerging technologies work consistently and accurately [37]. People who decide to adopt services based on emerging technology usually regard perceived reliability as a crucial factor [37]. Perceived reliability is critical to whether users decide to adopt mHealth apps. For example, if people believe that mHealth apps are reliable and may cause benefits, they will be willing to continue using them. Barua et al. [49] shed light on the effect of perceived reliability on users’ satisfaction with self-services technologies. Moreover, perceived reliability positively affects users’ satisfaction with mobile banking [50]. People are satisfied when they find that emerging technologies are reliable and safe [49,51,52].

**Hypothesis** **5** **(H5).** *Perceived reliability positively affects users’ e-satisfaction with mHealth apps*.

### 3.6. Price Value

The definition of price value is peoples’ perceived trade-off between their profit and cost in using IT [33]. When the perceived benefits of usage behavior exceed the monetary costs, price value will increase users’ satisfaction with mHealth apps. Using mHealth apps reduces the financial and non-financial costs of going to hospitals. For example, if physicians can provide health services through mHealth apps, the patient does not need to go to the hospital for services. This saves the patients and physicians financial costs and valuable time. People may be satisfied with the experience of using mHealth apps if relevant benefits are considered higher than financial costs.

**Hypothesis** **6** **(H6).** *Price value positively affects users’ e-satisfaction with mHealth apps*.

### 3.7. Online Review

Online reviews are word-of-mouth information on virtual platforms and are popular all over the world [53]. People are willing to read online reviews that have an influence on their consumption decisions [53,54]. The interactivity of mHealth apps allows users to create online reviews, feedback on health services, and attitudes on mHealth apps [55]. A significant correlation exists between online reviews and users’ willingness to use experience, such as entertainment and ease of use [56]. If users consider online reviews as valuable, useful, comprehensive, credible, and updated, they may be satisfied with mHealth apps [13].

**Hypothesis** **7** **(H7).** *Online review positively affects users’ e-satisfaction with mHealth apps*.

### 3.8. E-Satisfaction

E-satisfaction refers to how satisfied people are with their experience with an electronic commerce company [57]. Satisfaction reflects the degree to which users derive a positive attitude from a service experience [49,58]. E-satisfaction has an effect on the specific transaction based on the immediate usage experience, which may positively impact the continued intention of technologies adoption [59,60]. If the effects of using mHealth apps meet and exceed users’ expectations, they may be satisfied with mHealth apps and they might be more likely to intend to continue to use it. Users who are satisfied with mHealth apps may be motivated to continue using such apps. E-satisfaction stems from the difference between users’ expectations and the actual benefits of specific tools, and higher satisfaction may enhance people’s tendency to reuse these tools [59,61].

**Hypothesis** **8** **(H8).** *E-satisfaction positively affects users’ continued usage intention*.

### 3.9. Continued Usage Intention

Continued usage intentions refer to the degree of users’ perception of the willingness of continued usage behaviors [12,62]. In various fields, behaviors have been proven to be best predicted by behavioral intention [63]. Usage intentions and behaviors exist significant correlations, and continued usage behavior has been proved to be considerably affected by individuals’ intentions in the field of IT [62,64]. In addition, the usage intention positively affects the behavior of using mobile services in the healthcare field [12,60,65]. Continued usage intention predicted the behavior of using IT [66]. In the context of healthcare, users’ intention to keep using mHealth apps may predict the behavior of using these apps.

**Hypothesis** **9** **(H9).** *Continued usage intention positively affects the behavior of using mHealth apps*.

### 3.10. Habit

Habit refers to the extent to which people tend to carry out something automatically after a period of experience and reflects the result of previous usage experience [33,67]. Habit can be expressed as the tendency of users to act spontaneously because of their accumulated learning experience [67]. Habit reflects stable behaviors without active consideration and is repeatedly performed when the behaviors occur. When frequent behaviors are repeated to become habits, these behaviors are guided by automated cognitive processes rather than elaborate decision processing based on satisfaction and continued intentions [68]. In the context of healthcare, habit in mHealth apps use may predict the continued behavior of using these apps.

**Hypothesis** **10** **(H10).** *Habit positively affects continued behavior of using mHealth apps*.

Habit can be a basis for understanding the interaction between users and IT [33]. Habit refers to the tendency of users to take actions based on past experience [67]. Consumers’ habit in using mHealth apps for more pragmatic purposes rather than the novelty. Habit may impact users’ attitudes and beliefs that determine users’ intention of using mHealth apps and is one of the significant factors for mobile app acceptance [69,70,71]. The positive effect between e-satisfaction and continued intention may be affected by habits in mHealth apps use.

**Hypothesis** **11** **(H11).** *Habit positively moderates the effects of e-satisfaction on the continued intention of using mHealth apps*.

## 4. Methods

### 4.1. Development of Questionnaire

To guarantee the questionnaire validity and reliability, this study adapted items from previously validated instruments to use with the context of mHealth apps in China. We adopted the items from Venkatesh et al. [28,33] to measure performance expectancy, effort expectancy, social influence, and facilitating conditions. We used the items from Venkatesh et al. [33] and Alalwan [13] to examine price value, habit, and continued intention. We adapted the items from Alalwan [13] to measure online review and e-satisfaction. We adopted the measurement instruments from Mohammad et al. [12] to measure perceived reliability and continued usage behavior. We used a Likert seven–point scale to take the measurements of all items, which were listed in Appendix A.

Before an online survey, ambiguous items were checked through two stages: the expert review and the pilot study. In the first stage, five experts were invited to verify the measurement instruments whether fit the context of mHealth apps and review the instruments to see if they accurately reflected the construction of UTAUT2. All experts confirmed that the writing style and the feasibility of the measurement instruments of constructs have fit the context of mHealth apps and are accurate. In the second stage, we invited 20 interviewees to participate in a pilot study and modified ambiguous items based on some problems observed. We conducted an exploratory factor analysis to test the constructs. The results showed that the value of Kaiser–Meyer–Olkin (KMO) was 0.912 and Bartlett’s test of sphericity was significant. Finally, 46 items were included in the questionnaire.

The questionnaire consisted of three sections. First, the aim of this study was described, the conditions for participants to participate in the survey anonymously were described, and examples (Chunyuyisheng and Haodaifuzaixian) clarifying mHealth apps were provided. In addition, it was explained in the first part that only users who have previously used mHealth apps can participate in the survey. The second section is used for demographic issues, including age, gender, education level, and occupation. The last section is used to measure the 11 constructs in the research model.

### 4.2. Data Collection

We collected data through an online survey. All participants were required to have used mHealth apps. First, before issuing the questionnaire, we ensured that the participants had understood and used mHealth apps. Second, the questionnaire was online distributed through a Chinese online platform (Sojump). Sojump is the largest online survey platform in China, with 35.36 million users. Participants have reached Sojump and downloaded the questionnaire at the time of the researchers’ study. They have validly answered the researchers’ questions and voluntarily have no compensation or gifts to avoid potential biases. From March 2020 to June 2020, we completed the online survey by 380 smartphone users who have used mHealth apps. We excluded incomplete questionnaires and obtained 327 valid responses with a response rate of 86%.

We calculated the demographic characteristics, which are listed in Table 1. Of all respondents, 51.1% were females, while males accounted for 48.9% of all participants; 47.7% of participants were aged between 18 and 29 years, 40.1% of participants belonged to the 30–40 age group, and 12.2% were aged 40 years and above. Most participants (83.5%) in this study were college graduates. The majority of the participants’ experiences on mHealth apps usage were within 1 year (72.5%).

### 4.3. Data Analysis

To test the proposed hypotheses, this study conducted a three-step analysis method. First, this study used SPSS Statistics 25 to test the measurement model through confirmatory factor analysis. Second, this study used AMOS 26 to test the structural model through the structural equation model approach [72]. Third, this study used the bootstrapping analysis method to test the mediating role of e-satisfaction and the hierarchical regression method to test the moderating effect of habit [73].

## 5. Results

### 5.1. Measurement Model Analysis

The measurement model comes from the structural equation model and consists of the measurement instruments and constructs. The means and standard deviations of all constructs are listed in Table 2. We examine the reliability, content validity, and structural validity of each construct based on the sample examination. We conduct a confirmatory factor analysis. Since all the scales are based on existing studies, the validity of the questionnaire content is ensured. Cronbach’s alpha and composite reliability both exceed 0.7, which supports the reliability of the questionnaire [74]. Moreover, all factor loadings over 0.7 and the results reflect the good convergent validity of our scales [74]. All constructs’ average variance extracted (AVE) exceeded the acceptable value.

Discriminant validity is the degree to which measurement instruments are uncorrelated with other different constructs. Discriminant validity is exhibited if constructs’ square roots of AVE are greater than the correlation coefficient of the construct with any other construct [75]. Table 3 shows the good discriminant validity of the constructs. To test the model fit of the research model, we measure the fit indices and the results are as shown in Table 4 that indicates the goodness of fit assessments for the research model. In addition, this study used self-reported data, which may cause common method bias [76]. To investigate common method bias, this study conducts Harmon’s one–factor test and found that the single factor could explain 37% of the total variance. Thus, this study does not have a common method bias.

### 5.2. Structural Model Analysis

**Figure 2:**
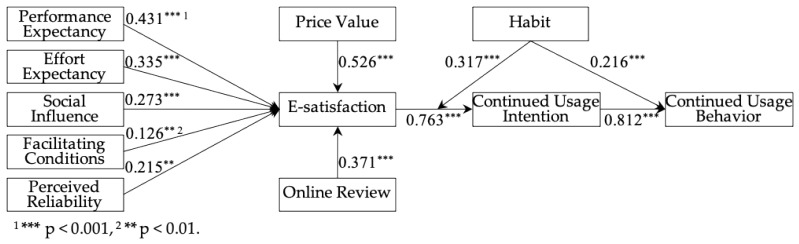
Results of the hypotheses testing.

The results of the hypothesis testing, including standardized path coefficients, and the amount of variance explained are shown in Table 5 and Figure 2. The results show that our hypotheses were supported. The relationship between performance expectancy and continued usage intention was found to be statistically significant, thereby supporting H1. The results reveal the significant impact of effort expectancy, social influence, facilitating conditions, perceived reliability, price value, online review on users’ continued intention of using mHealth apps. Thus, this study identified the effects of antecedents on continued usage intention, and H2, H3, H4, H5, H6, and H7 were supported. Moreover, this study found that e-satisfaction positively affects continued usage intention and H8 was supported. Consistent with existing studies [12], this study also verified the positive relationship between behavioral intention and usage behavior. In addition, as displayed on Table 5, we found a moderation effect of habit on the relationship between continued usage intention and users’ e-satisfaction with mHealth apps. Thus, H11 was supported.

**Figure 3:**
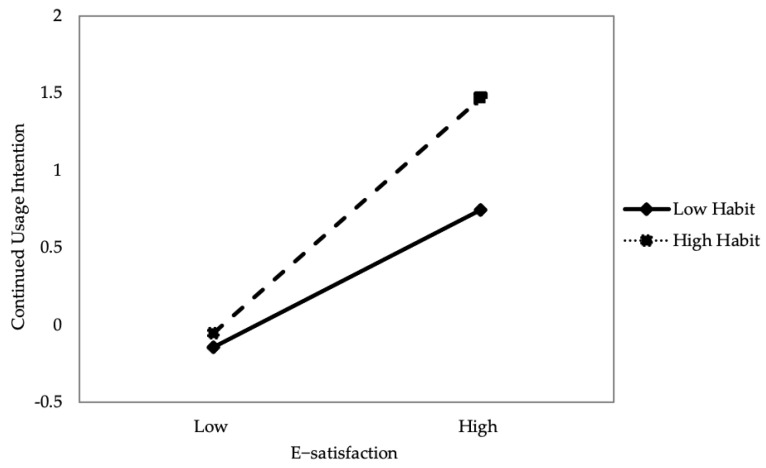
Results of the moderation effect.

This study examined the mediating model using 5000 bootstrap samples, which has been proven to be more accurate than the Sobel test [73,77]. As shown in Table 6, all indirect effects are significant and the 95% confidence interval for the estimates of the mediation effects excludes 0 [27]. Thus, the bootstrapping results showed that the mediation effects of e-satisfaction were significant. In addition, we used the hierarchical regression method to test the moderation effect. As shown in Table 5 and Figure 3, the higher habit will strengthen the positive relationship between user e-satisfaction and continued usage intention.

## 6. Discussion

This study aims to explore the influences of users’ continued behaviors of mHealth apps usage from the perspective of UTAUT2. Our results proved that performance expectancy, social influence, facilitating conditions, perceived reliability, price value, online review have significant positive impacts on users’ continued usage intention through the mediation role of e-satisfaction. Moreover, users’ continued intention positively impacts the usage behavior of keeping to use mHealth apps. It is further identified that habits of using mHealth apps enhance the positive effect of e-satisfaction on continued usage intention.

This study identifies that habit directly affects users’ continuous use of mHealth apps and users should be attracted to form the habit of mHealth apps usage, which is crucial to these apps’ successful development. Interestingly, the stronger the habit of using mHealth apps, the stronger the relationship between users’ e-satisfaction and their willingness to continue using mHealth apps. Generally, individuals who are satisfied with experience tend to repeat the behavior. Furthermore, individuals who develop habits towards emerging technologies are likely to continue to use the apps.

Two constructs (perceived reliability and online review) added to the research model have important effects on the continued intention of using mHealth apps. For example, users are significantly interested in the availability of online comments provided by others in mHealth apps. This suggests that users regard such comments as reliable, useful, and relevant health sources in these platforms when consulting professional physicians. Thus, extant reviews published by others can be easily accessed and conveniently through mHealth apps. In addition, continued behavior of mHealth apps usage also can facilitate users’ seeking health information, thereby saving their time and effort. In this study, the empirical results show that all hypotheses have been verified and the extended UTAUT2 is an important theoretical model for predicting the continued use of mHealth apps.

### 6.1. Theoretical Implications

Because mHealth apps are an emerging technology in China, a deeper understanding of the usage of these apps is required. Our purpose is to explore the effects of users’ e-satisfaction with mHealth apps and actual usage behavior from the perspective of the extended UTAUT2. There are some theoretical implications in the study.

First, this study pays attention to users’ continued acceptance of mHealth apps. Prior studies mainly examined the effect of users’ initial behavioral intention of emerging technologies while ignoring continuous usage behaviors. However, uses’ continuous intention and usage behavior are significantly important for the success of emerging technologies. This study extends the UTAUT2 in the field of continuous use of mHealth apps.

Second, this study contributes by validating the effects of perceived reliability and online review on e-satisfaction with mHealth apps. In the context of healthcare services, due to the life-threatening possibilities, perceived reliability is important for their selection. In the context of mobile apps, people believe that online reviews are valuable sources of information when they are selecting services and products. Thus, in these two respects, this study integrates perceived reliability and online reviews into the UTAUT2 model, which enriches the theoretical framework.

Third, the key strength of this study is revealing the moderating role of habits. The results reveal that when people are accustomed to using mobile apps, then the relationship between e-satisfaction and continued intention will be enhanced. Habit has the moderating effect between e-satisfaction and continued intention of using mHealth apps, and this finding enriches the research on mHealth apps and expands the theoretical scope of UTAUT2.

### 6.2. Practical Implications

The study has some practical implications regarding the understanding of designing and managing mHealth apps. First, managers of mHealth apps should organize promotional activities and pay attention to the roles of performance expectation and effort expectation. Users of mHealth apps should be encouraged to use mHealth apps due to requiring less time and effort compared to the traditional ways of waiting and visiting hospitals. In addition, the price value of health services on mHealth apps positively affects continued usage intention through the mediation role of e-satisfaction. In this study, we suggest that managers should regulate the price value of health services on mHealth apps.

Second, managers should encourage users to publish rich online reviews on the features and health services of mHealth apps. For example, the number of users who review their attitude of the experience in such apps should be a focus. To ensure that online review is relevant and credible to other users, mHealth apps should improve the information quality of the published online reviews [53], which convinces users that online reviews are valuable to acquire health services and information. In addition, this study found that users’ habits moderated the positive relationship between e-satisfaction and continued intention of using mHealth apps. Users’ habits can be enhanced by personalized notifications of using mHealth apps [78].

Third, managers of mHealth apps could focus on increasing users’ e-satisfaction with the functions of mobile platforms, thereby improving users’ intention of continues using mHealth apps to seek health information and services. In addition, if users receive high-quality and friendly personalized healthcare services, then they will be satisfied with their experience and the high value of using such apps. Regarding mHealth apps that have the ability to capture and record large amounts of patient data, physicians could implement and provide personalized health services through these mobile platforms. Moreover, users have autonomous control in the processes of consulting and can provide appropriate and relevant solutions to problems in mHealth apps.

### 6.3. Limitations and Future Research Directions

Though this study is dedicated to providing insights into using mHealth apps, there are also some noted limitations. First, due to the nature of cross-sectional research, this study could not accurately explain users’ perceptions of mHealth apps within the time frame. Future studies should conduct longitudinal research to discuss users’ perceptions and attitudes toward mHealth apps over time. Second, in the current study, to draw conclusions from this special subgroup for the entire Chinese group of 18–70-year-olds who use the mHealth apps is a limitation. In future research, other types of sampling techniques for collecting data should be considered to apply. Third, though amounts of constructs have been considered in the current study, other constructs, such as health service quality, doctor-patient interaction, and individuation, should be studied. In addition, personality trait constructs, such as self-efficacy, e-health literacy, and health consciousness, should be considered, as this might shed light on the insights of supporting mHealth apps usage.

## 7. Conclusions

Considering the particularities of mHealth apps, we propose a research model based on the appropriate theoretical model that extends two factors, perceived reliability and online review, to the original UTAUT2 to explore the effects of factors on the continued behavior of using mHealth apps. Our results prove that performance expectancy, social influence, facilitating conditions, perceived reliability, price value, online review have significant positive impacts on users’ continued usage intention through the mediation role of e-satisfaction. Moreover, users’ continued intention positively impacts the usage behavior of keeping to use mHealth apps. It is further identified that habits of using mHealth apps enhance the positive effect of e-satisfaction on continued usage intention. Finally, we express theoretical and practical implications and discuss the limitations and future research directions.

## Figures and Tables

**Table 1:** Demographic profiles of samples (N = 327).

Demographic Profile	Frequency	Percentage (%)
Gender		
Male	160	48.9
Female	167	51.1
Age		
18–29	156	47.7
30–39	131	40.1
40 and above	40	12.2
Level of education		
High school and below	20	6.1
College graduate	273	83.5
Postgraduate and above	34	10.4
Experience in using mHealth apps		
1 years and below	237	72.5
1–3 years	76	23.2
3 years and above	14	4.3

**Table 2:** Results of Constructs Validity and Reliability.

Construct	Item	Mean	SD ^12^	Factor Loading	Cronbach’s Alpha	CR ^13^	AVE ^14^
PE ^1^	PE1	5.917	0.834	0.761	0.750	0.839	0.567
	PE2	5.404	1.128	0.699			
	PE3	5.700	1.103	0.735			
	PE4	5.572	1.352	0.813			
EE ^2^	EE1	5.823	1.131	0.742	0.760	0.831	0.552
	EE2	5.147	1.532	0.814			
	EE3	5.697	1.336	0.692			
	EE4	5.648	1.115	0.719			
SI ^3^	SI1	5.208	1.571	0.798	0.800	0.868	0.622
	SI2	5.067	1.506	0.766			
	SI3	5.128	1.598	0.770			
	SI4	5.313	1.420	0.819			
FC ^4^	FC1	5.624	1.745	0.690	0.744	0.837	0.563
	FC2	5.547	1.440	0.752			
	FC3	5.297	1.218	0.820			
	FC4	5.425	1.302	0.733			
PR ^5^	PR1	5.517	1.149	0.703	0.767	0.860	0.607
	PR2	5.005	1.298	0.776			
	PR3	5.244	1.101	0.853			
	PR4	5.560	0.999	0.777			
PV ^6^	PV1	5.358	1.233	0.765	0.780	0.841	0.799
	PV2	5.413	1.135	0.759			
	PV3	5.495	1.198	0.869			
ORE ^7^	ORE1	5.323	1.210	0.761	0.852	0.912	0.597
	ORE2	5.294	1.174	0.730			
	ORE3	4.917	1.617	0.716			
	ORE4	5.321	1.331	0.801			
	ORE5	5.165	1.367	0.745			
	ORE6	5.171	1.512	0.862			
	ORE7	5.596	1.250	0.786			
ESA ^8^	ESA1	5.670	0.992	0.722	0.784	0.852	0.590
	ESA2	5.294	1.296	0.746			
	ESA3	5.498	1.125	0.846			
	ESA4	5.495	1.247	0.753			
CUI ^9^	CUI1	5.853	1.011	0.745	0.809	0.789	0.556
	CUI2	5.765	1.214	0.758			
	CUI3	5.627	1.378	0.733			
CUB ^10^	CUB1	5.428	1.381	0.718	0.724	0.775	0.535
	CUB2	5.356	1.482	0.752			
	CUB3	5.480	1.244	0.723			
HAB ^11^	HAB1	5.064	1.724	0.820	0.853	0.871	0.629
	HAB2	4.957	1.265	0.751			
	HAB3	5.076	1.352	0.788			
	HAB4	4.971	1.686	0.811			

^1^ Performance Expectancy, ^2^ Effort Expectancy, ^3^ Social Influence, ^4^ Facilitating Conditions, ^5^ Perceived Reliability, ^6^ Price Value, ^7^ Online Review, ^8^ E-satisfaction, ^9^ Continued Usage Intention, ^10^ Continued Usage Behavior, ^11^ Habit, ^12^ Standard Deviation, ^13^ Composite Reliability, ^14^ Average Variance Extraction.

**Table 3:** Discriminant Validity.

	PE	EE	SI	FC	PR	PV	ORE	ESA	CUI	CUB	HAB
PE ^1^	0.757 ^12^										
EE ^2^	0.528 ^13^	0.743									
SI ^3^	0.677	0.676	0.789								
FC ^4^	0.561	0.461	0.514	0.750							
PR ^5^	0.441	0.253	0.261	0.409	0.779						
PV ^6^	0.673	0.581	0.681	0.391	0.303	0.799					
ORE ^7^	0.507	0.339	0.481	0.243	0.187	0.549	0.773				
ESA ^8^	0.685	0.518	0.626	0.537	0.335	0.594	0.494	0.768			
CUI ^9^	0.666	0.597	0.645	0.468	0.412	0.684	0.480	0.571	0.745		
CUB ^10^	0.452	0.358	0.505	0.225	0.216	0.626	0.451	0.440	0.536	0.731	
HAB ^11^	0.005	0.024	0.087	0.066	0.066	0.195	0.123	0.006	0.033	0.059	0.793

^1^ Performance Expectancy, ^2^ Effort Expectancy, ^3^ Social Influence, ^4^ Facilitating Conditions, ^5^ Perceived Reliability, ^6^ Price Value, ^7^ Online Review, ^8^ E-satisfaction, ^9^ Continued Usage Intention, ^10^ Continued Usage Behavior, ^11^ Habit, ^12^ Diagonal values are squared roots of AVE, ^13^ Off-diagonal values are the estimates of inter-correlation between the latent constructs.

**Table 4:** Goodness of fit assessments for the research model.

Goodness of Fit Measures	CMIN/DF ^1^	IFI ^2^	TLI ^3^	CFI ^4^	RMSEA ^5^
Goodness of fit ranges	1–3	>0.900	>0.900	>0.900	<0.050
Model fit	1.135	0.985	0.981	0.985	0.020

^1^ Chi square/Degrees of freedom, ^2^ Incremental fit index, ^3^ Tucker–Lewis index, ^4^ Comparative fit index, ^5^ Root-mean-square error of approximation.

**Table 5:** Results of the Hypotheses Testing.

Hypothesis	Relationship	Std. Beta	Std. Error	*t*-Value	Result
H1	PE ^1^→ESA	0.431 *** ^12^	0.063	7.254	Support
H2	EE ^2^→ESA	0.335 ***	0.047	7.128	Support
H3	SI ^3^→ESA	0.273 ***	0.051	5.352	Support
H4	FC ^4^→ESA	0.126 ** ^13^	0.036	3.315	Support
H5	PR ^5^→ESA	0.215 **	0.068	4.357	Support
H6	PV ^6^→ESA	0.526 ***	0.071	7.408	Support
H7	ORE ^7^→ESA	0.371 ***	0.055	6.745	Support
H8	ESA ^8^→CUI	0.763 ***	0.092	8.293	Support
H9	CUI ^9^→CUB ^10^	0.812 ***	0.101	8.039	Support
H10	HAB ^1^^1^→CUB	0.216 ***	0.039	5.538	Support
H11	HAB and ESA→CUI	0.317 ***	0.056	5.661	Support

^1^ Performance Expectancy, ^2^ Effort Expectancy, ^3^ Social Influence, ^4^ Facilitating Conditions, ^5^ Perceived Reliability, ^6^ Price Value, ^7^ Online Review, ^8^ E-satisfaction, ^9^ Continued Usage Intention, ^10^ Continued Usage Behavior, ^11^ Habit, ^12^ *** *p* < 0.001, ^13^ ** *p* < 0.01.

**Table 6:** Bootstrapping analysis of the mediation effect of e-satisfaction.

Effects	Dependent Variable	CUI ^8^	Effects	Boot SE	Bootstrap 95% CI	
					Boot LLCI	Boot ULCI
Total Effects	Independent Variables	PE ^1^	0.532 *** ^9^	0.080	0.378	0.686
		EE ^2^	0.494 ***	0.047	0.405	0.588
		SI ^3^	0.345 ***	0.045	0.256	0.433
		FC ^4^	0.465 ***	0.045	0.383	0.556
		PR ^5^	0.473 ***	0.043	0.391	0.560
		PV ^6^	0.686 ***	0.057	0.577	0.802
		ORE ^7^	0.308 ***	0.024	0.261	0.355
Indirect Effects	Independent Variables	PE	0.215 ***	0.043	0.081	0.370
		EE	0.229 ***	0.037	0.133	0.322
		SI	0.095 ** ^10^	0.033	0.032	0.160
		FC	0.189 ***	0.037	0.105	0.279
		PR	0.191 ***	0.038	0.113	0.271
		PV	0.346 ***	0.051	0.222	0.473
		ORE	0.134 ***	0.027	0.081	0.187
Direct Effects	Independent Variables	PE	0.683 ***	0.045	0.532	0.849
		EE	0.589 ***	0.043	0.490	0.697
		SI	0.473 ***	0.051	0.372	0.574
		FC	0.581 ***	0.043	0.489	0.669
		PR	0.605 ***	0.041	0.513	0.699
		PV	0.850 ***	0.050	0.741	0.959
		ORE	0.410 ***	0.024	0.363	0.457

^1^ Performance Expectancy, ^2^ Effort Expectancy, ^3^ Social Influence, ^4^ Facilitating Conditions, ^5^ Perceived Reliability, ^6^ Price Value, ^7^ Online Review, ^8^ Continued Usage Intention, ^9^ *** *p* < 0.001, ^10^ ** *p* < 0.01.

## Data Availability

The data presented in this study are available on request from the corresponding author.

## Appendix A. Measurement Items

**Table A1:** Measurement items of constructs.

Constructs	Items	Statements	Sources
Performance Expectancy (PE)	PE1	I think mHealth apps is helpful for my health.	[28,33]
	PE2	I think mHealth apps could solve individual health problems.	
	PE3	I think mHealth apps can manage individual health quickly.	
	PE4	I think mHealth apps can increase the capability of health self-management.	
Effort Expectancy (EE)	EE1	I think I can easily learn to use mHealth apps.	[28,33]
	EE2	I can understand the health information on mHealth apps.	
	EE3	I can easily use mHealth apps.	
	EE4	I can get the skill of using mHealth apps.	
Social Influence (SI)	SI1	I would adopt mHealth apps based on friends’ and relatives’ perspectives.	[28,33]
	SI2	I would adopt mHealth apps based on individuals who influence my behavior.	
	SI3	I would adopt mHealth apps based on friends and relatives.	
	SI4	Using mHealth apps is more prestigious than not using them.	
Facilitating Conditions (FC)	FC1	I possess the resources needed to accept mHealth apps.	[28,33]
	FC2	I possess the knowledge needed to accept mHealth apps.	
	FC3	The adoption of technologies is consistent with my others.	
	FC4	I can acquire helps from others when I account for problems.	
Perceived Reliability (PR)	PR1	I can get exact and true health information in mHealth apps.	[12]
	PR2	I depend on the health information through mHealth apps.	
	PR3	I think mHealth apps are persistent.	
	PR4	I think mHealth apps keep criterion constantly.	
Price Value (PV)	PV1	I can get knowledge at a rational price through these apps.	[13,33]
	PV2	Health services in mHealth apps are fine price for money.	
	PV3	mHealth apps offer worth for users in price.	
Online Review (ORE)	ORE1	Online reviews in mHealth apps are believable.	[13]
	ORE2	Online reviews in mHealth apps are relevant to my demands.	
	ORE3	Online reviews in mHealth apps are trusted.	
	ORE4	Online reviews in mHealth apps have adequate deepness.	
	ORE5	Online reviews in mHealth apps have adequate broadness.	
	ORE6	Online reviews’ quantities can satisfy my health demands.	
	ORE7	Online reviews are useful to assess health information.	
E-satisfaction (ESA)	ESA1	I am normally willing to adopt mHealth apps.	[13]
	ESA2	I am extremely pleased with mHealth apps.	
	ESA3	I am joyful to adopt mHealth apps.	
	ESA4	I am pleased with the business dealings mHealth apps.	
Habit (HAB)	HAB1	The use of mHealth apps turn into my custom.	[13,33]
	HAB2	I am immersed in accepting mHealth apps.	
	HAB3	I have to adopt mHealth apps.	
	HAB4	The adoption of mHealth apps has become intrinsic behavior.	
Continued Usage Intention (CUI)	CUI1	I prepare to continue accepting mHealth apps in the future.	[13,33]
	CUI2	I always accept mHealth apps in daily life.	
	CUI3	I purpose to continue accepting mHealth apps frequently.	
Continued Usage Behavior (CUB)	CUB1	I continue to go through much time in using mHealth apps.	[12,33]
	CUB2	I desire mHealth apps to keep my health safe in the future.	
	CUB3	I use mHealth apps on regular basis in the future.	
